# Short Warwick-Edinburgh Mental Well-being Scale (SWEMWBS): performance in a clinical sample in relation to PHQ-9 and GAD-7

**DOI:** 10.1186/s12955-021-01882-x

**Published:** 2021-11-24

**Authors:** Neha Shah, Mizaya Cader, Bill Andrews, Rose McCabe, Sarah L. Stewart-Brown

**Affiliations:** 1 City University London, London, UK; 2 National Dengue Control Unit, Ministry of Health, Colombo, Sri Lanka; 3 Department of Psychology, University of Roehampton, London, UK; 4 Emeritus Professor of Public Health, Warwick Medical School University of Warwick, Coventry, UK

**Keywords:** SWEMWBS, PHQ-9, GAD-7 mental wellbeing, Anxiety, Depression, Outcome measure, Counselling, Primary care, Clinical

## Abstract

**Purpose:**

This study assesses the construct validity and sensitivity to change of the Short Warwick-Edinburgh Mental Well-being Scale (SWEMWBS) as an outcome measure in the treatment of common mental disorders (CMD) in primary care settings.

**Methods:**

127 participants attending up to 5 sessions of therapy for CMD in primary care self-rated the SWEMWBS, the Patient Health Questionnaire (PHQ-9) and General Anxiety Disorder (GAD-7) scales. SWEMWBS’s construct validity and sensitivity to change was evaluated against the PHQ-9 and GAD-7 across multiple time points in two ways: correlation coefficients were calculated between the measures at each time point; and sensitivity to change over time was assessed using repeated measures ANOVA.

**Results:**

Score distributions on SWEMWBS, but not PHQ-9 and GAD-7, met criteria for normality. At baseline, 92.9% (118/127) of participants scored above clinical threshold on either PHQ-9 or GAD-7. Correlations between SWEMWBS and PHQ-9 scores were calculated at each respective time point and ranged from 0.601 to 0.793. Correlations between SWEMWBS and GAD-7 scores were calculated similarly and ranged from 0.630 to 0.743. Significant improvements were seen on all three scales over time. Changes in PHQ-9 and GAD-7 were curvilinear with greatest improvement between sessions 1 and 2. Change in SWEMWBS was linear over the five sessions.

**Conclusions:**

This exploratory study suggests that SWEMWBS is acceptable as a CMD outcome measure in primary care settings, both in terms of construct validity and sensitivity to change. Given patient preference for positively over negatively framed measures and statistical advantages of measures which are normally distributed, SWEMWBS could be used as an alternative to PHQ-9 and GAD-7 in monitoring and evaluating CMD treatment.

## Introduction

The Warwick-Edinburgh Mental Well-Being Scale (WEMWBS) was developed in 2007 [1] to support the emerging discipline of public mental health [2] by enabling monitoring of positive mental health and evaluation of interventions, programmes and approaches to promoting mental health particularly in non-clinical populations. Development involved qualitative research with members of the general public and mental health service users regarding the nature of mental wellbeing as well as the face validity of the scale [1]. In 2009, a seven item version of the original 14 item scale, the Short Warwick–Edinburgh Mental Well-Being Scale (SWEMWBS), was developed using Rasch modelling to provide better fit as an interval scale measure of mental wellbeing [3]. The SWEMWBS also has the added benefit of being shorter and thus less onerous to complete.

The concept of mental wellbeing was developed in the context of positive psychology [4]. It is now recognised as a core indicator of overall health in some jurisdictions [5] and some countries now use (S)WEMWBS as an indicator in this context [6–10]. The concept has proved of interest in both mental health services and primary care, where it has been linked to patient-centred and recovery agendas [11–15]. The best approach to measurement of mental wellbeing has been widely debated, but consensus is growing to support the conceptual framework within which WEMWBS was developed, in which mental wellbeing covers aspects of both feeling good (Hedonia) and functioning well (Eudaimonia) [16, 17]. The SWEMWBS also covers both concepts although has fewer items relating to feeling good than functioning well [3]. Some have proposed a ‘dual continuum’ model in which mental illness and mental wellbeing represent different dimensions and thus should be measured separately [18], whereas others perceive mental wellbeing and mental ill-health to represent extremes of a single underlying factor. Whilst there is no consensus on single/dual continuum hypotheses, studies to date show a high degree of (inverse) correlation between WEMWBS and widely respected measures of mental illness [1, 19].

Common mental health disorders (CMDs) comprise of depression, anxiety, panic attacks, and obsessive compulsive disorders and are highly prevalent being experienced by 14.7% of men and 23.1% of women in the UK according to 2014 estimates [20]. CMDs affect people’s ability to work, care for others and participate in society. Lost productivity as a result of two of the most common mental disorders, anxiety and depression costs the global economy US$ 1 trillion each year [21]. Only 39% of people with CMD in the UK report accessing treatment [20], meaning there is a need to explore the effectiveness of non-specialist mental health interventions in community, primary care, and public health settings for this group as well as increasing access to clinical intervention. The recent COVID pandemic has been associated with increases in estimated prevalence of CMD, and the longer term impacts of this are yet to be seen [22].

Qualitative studies have indicated that mental health service users and their carers would choose WEMWBS over other commonly used measures for monitoring and evaluating the effect of interventions on their mental health [23]. WEMWBS scored higher on appropriateness rating scales than the PHQ-9 [24] with users highlighting a preference for scales that are positively framed and reflect the judgements and interests of service users over those of professionals [23]. As a result the scales are now being used in NHS and private health services often in conjunction with other measures of mental illness such as the PHQ-9 and GAD-7, and the CORE Outcome Measure [25, 26]. It has become important to investigate the validity of both WEMWBS and SWEMWBS for use as outcome and monitoring measures in people with CMD to address the burden of CMD in more appropriate ways and to allow comparison of approaches across settings. Studies in different settings suggest these measures are valid in psychiatric populations [13, 27, 28] and primary care [29], but comparative performance in relation to commonly used clinical measures at multiple time points during a course of treatment has not been investigated.

The aim of this study was to investigate the construct validity of SWEMWBS as an outcome measure in patients with CMD undergoing psychological therapy in comparison to two widely used primary care based clinical outcome measures, the Patient Health Questionnaire 9 (PHQ-9) [30] and the General Anxiety Disorder 7 scale (GAD-7) [31].

## Methods

### Data collection

The data for this study were gathered using electronic software developed by the Pragmatic Research Network, a collaboration of professionals promoting service-based evaluation and feedback-informed treatment. The software allows session by session administration of outcome measures, producing reports for feedback to clients and therapists to inform the progress of the therapy and service evaluation.

Data were collected by therapists practicing a form of psychotherapy, cognitive hypnotherapy (CHT). Therapists were informed about the proposed study through an online forum and invited to participate in the research. Fully anonymised data from consented participants and therapists was made available for the study. The Pragmatic Research Network supervised data collection for those who consented to take part, providing an initial training day and a combination of face-to-face, telephone and e-mail support to ensure an appropriate standard was reached.

CHT uses induction into a trance-like state to access unconscious problematic thoughts, feelings, and memory patterns. Therapy focuses on interrupting faulty pattern matching by changing the context, structure, process or consequence of the problem pattern and is individually tailored to clients’ needs [32]. It starts with an initial session which seeks to introduce the client to hypnosis, uncover the language clients use and identify the unconscious phenomena they experience whilst acting within unconscious patterns. As such the therapeutic process begins during the first session. The three measures were administered before each therapeutic session including the first and discussed within the sessions to provide feedback and inform progress. The length and frequency of subsequent treatment is negotiated between therapist and client based upon progress, ongoing need, and willingness to pay. A low number of sessions may indicate early success of treatment, termination due to perceived ineffectiveness or not liking the process, or limited willingness to pay for the therapy [32].

### Participants

Participants were adults seeking CHT as treatment for mental health problems, mainly anxiety and depression, at the private, fee paying practices of participating therapists between October 2014 and April 2016. All participants were informed about the research objectives and written consent was obtained. 127 participants were recruited and provided data at the first session (Therapy 1) and at least one more session, enabling measurement of change over time; 34 participants provided data for five consecutive sessions.

### Measures

Participants completed three measures for the first time before Therapy 1 and each subsequent treatment session. Measures were given to participants to complete using the web based ‘pragmatic tracker’ software either via email link or on arrival to their session. The three measures were the SWEMWBS [3]; PHQ-9 [24] and GAD-7 [31]. Both GAD-7 and PHQ-9 are measures of CMD developed for evaluation of the UK Increasing Access to Psychological Therapy programme [33] and now widely used in primary care.

SWEMWBS is a seven item self-report measure of mental wellbeing [3]. Construct validity has been confirmed in diverse populations [3, 10, 28, 34–36] using methods such as confirmatory factor analysis and Rasch modelling, alongside assessment of external validity in comparison to other scales and content validity. Test–retest reliability has been confirmed in other populations [37]. SWEMWBS is responsive to change at group (standard response mean (SRM) ranged from 0.49 to 1.01 and probability of change statistic from 0.65 to 0.88) and individual level (at the threshold 2.77 standard errors of the mean (SEM) SWEMWBS demonstrated change in 20.1–80.6% of participants) [38]. The England population mean for SWEMWBS is (23.6) and cut points of one standard deviation above (28.1) and below (19.3) the mean have been used to derive normal, low and high levels of mental wellbeing respectively [35]. SWEMWBS has been translated into various languages [39].

PHQ-9 is a self-administered version of Primary Care Evaluation of Mental Disorders (PRIME-MD), an instrument developed to assist primary care clinicians in making criteria-based diagnoses of five types of Diagnostic and Statistical Manual of Mental Disorders, 4^th^ edition (DSM-IV) disorders, mood, anxiety, somatoform, alcohol, and eating (the current manual at time of development). The nine item PHQ-9 is the depression module of the full PHQ and consists of the nine criteria on which the diagnosis of DSM-IV depressive disorders is based [40]. PHQ-9 scores of greater than or equal to 5, 10, 15, and 20 out of 27 represent mild, moderate, moderately severe, and severe depression, respectively. PHQ-9 has been widely used as a diagnostic tool as well as a measure of depression severity, and has been shown to be responsive to change with a minimum clinically important difference of 5 points based upon a difference of 2 standard errors of the mean (SEM) in a clinical sample [30, 41].

The GAD-7 is a self-rated tool for screening for generalised anxiety disorder (GAD) and assessing its severity in clinical practice and research. Initial items were based on DSM-IV diagnostic symptom criteria for GAD and existing anxiety scales. The seven item anxiety scale (GAD-7) has been shown to have good reliability as well as criterion, construct and factorial validity when administered in primary care settings [31]. Cut off scores of greater than or equal to 5, 10, and 15 out of 21 represent mild, moderate and severe anxiety respectively, and a clinical cut off score of 10 has been identified as indicating probable diagnosis of GAD, social phobia, panic disorder, and posttraumatic stress disorder based on receiver operator curve (ROC) analyses. When used in a clinical sample the scale demonstrates ability to detect change with a moderate pre-post treatment effect size (Cohen’s D) of 0.78 [42].

### Statistical analysis

Analyses were performed using SPSS 24.0. Score distributions for each scale were assessed by visual inspection and the Shapiro–Wilk test for normality. Mean and standard deviation (SD) scores for SWEMWBS, PHQ-9 and GAD-7 were calculated at baseline (assessment) and at each subsequent time point. A paired samples *T*-test was used to assess change from baseline to last measurement for participants who completed two, three, four and five sessions respectively.

### Correlation

Spearman’s Rho was used to assess the correlation between the measures at different time points.

### SWEMWBS measurement properties

Internal consistency was calculated for SWEMWBS in this clinical sample using Cronbach’s Alpha at Time 1 and Time 5.

### Time series analysis

Time series analyses were conducted (using General Linear Model with Repeated Measures) to assess significance of change over time for participants attending two, three, four and five sessions respectively across each clinical outcome measure. Within subject effects tests were carried out to assess difference of scores over repeated sessions, and further within subject contrast tests were carried out where data for more than two time points were available to identify polynomial contrasts (whether the trend was linear, quadratic, cubic or alternate mathematical pattern). Corrections were applied where the data did not comply with the assumption of sphericity, in order to maintain the power of the test and reduce the chance of type 2 (false negative) errors. Greenhouse–geisser corrections were applied when Mauchly’s test of sphericity resulted in ε < 0.75 and Huynh Feldt when Mauchly’s test of sphericity resulted in ε > 0.75 [43]

## Results

### Descriptive

The mean age of the participants (N = 127) was 38.0 (95% CI 35.4–40.6) years; 74.8% (N = 95) were female and 96.1% (N = 105) White. The majority were employed, lived with a partner and not on medication (Table 1). Data was collected on participating therapists and clients only and the overall consent rate is unknown. For 86.6% (N = 110) PHQ-9 scores exceeded clinical threshold for depression and for 89.0% (N = 113) of participants GAD-7 scores exceeded clinical threshold for anxiety at Therapy Session 1. Most participants appeared to have a mixed picture of anxiety and depression symptoms, 82.6% (N = 105) exceeded clinical threshold for both scales. For 70.1% (N = 89) SWEMWBS scores were at or below the 1 SD cut point for low mental wellbeing of 19.3 at Therapy session 1.

**Table 1:** Demographic characteristics

Demographic characteristic	Category, N (%)				
Gender	Female	Male	Not specified		
	95 (74.8%)	28 (22%)	4 (3.1%)		
Age	18–29	30–49	50–69	70 +	Not specified
	22 (17.3%)	63 (49.6%)	19 (15.0%)	2 (1.6%)	6 (4.7%)
Ethnicity	White	African	Indian	Mixed-ethnicity	Not specified
	105 (96.1%)	1 (0.8%)	6 (4.7%)	1 (0.8%)	14 (11%)
Employment	Employed	Unemployed			
	100 (78.7%)	27 (21.3%)			
Living arrangements	Alone	With partner	With parents	Other shared	Not Specified
	25 (19%)	81 (63.8%)	15 (11.8%)	5 (3.9%)	1 (0.8%)
Medication	Antidepressants	Anxiolytics/hypnotics	Other	None	Not specified
	17 (13.4%)	2 (1.6%)	5 (3.9%)	99 (78.0%)	4 (3.1%)

127 participants attended and provided data at two therapy sessions, 94 at three, 63 at four and 34 at five. Mean scores are shown in Table 2. Participants who attended more than two sessions had slightly lower SWEMWBS scores at baseline (five sessions 18.8; four sessions 18.6; and three sessions 18.6 cf 19.0 for one session only) and higher PHQ-9 (five sessions 13.0; four sessions 12.2 and three sessions 12.2 cf 11.5 for one session only) and GAD-7 (five sessions 13.1; four sessions 12.8 and three sessions 12.1 cf 11.6 for one session only) scores at baseline, indicating worse states of mental health at start of therapy.

**Table 2:** Mean and standard deviation of scores for SWEMWBS PHQ-9 and GAD-7 at all time points

Participants group (N)	Time	SWEMWBS mean score (SD)	PHQ-9 mean score (SD)	GAD-7 mean score (SD)
Participants who completed two sessions (N = 127)	Therapy 1	19.0 (3.6)	11.5 (6.2)	11.6 (5.4)
	Therapy 2	20.4 (4.1)	7.8 (6.2)	8.3 (5.5)
	Change 1–2	1.4 (2.9) *P* < 0.005	− 3.7 (4.7) *P* < 0.005	− 3.3 (3.8) *P* < 0.005
Participants who completed three sessions (N = 94)	Therapy 1	18.6 (3.6)	12.2 (6.0)	12.1 (5.6)
	Therapy 2	20.0 (4.0)	8.4 (5.9)	8.9 (5.6)
	Therapy 3	21.6 (5.4)	6.9 (6.0)	7.1 (5.5)
	Change 1–3	3.0 (4.4) *P* < 0.005	− 5.3 (5.9) *P* < 0.005	− 5.1 (5.2) *P* < 0.005
Participants who completed four sessions (N = 63)	Therapy 1	18.6 (3.7)	12.2 (6.0)	12.8 (5.1)
	Therapy 2	19.9 (3.5)	8.5 (5.7)	9.1 (5.5)
	Therapy 3	21.2 (4.3)	7.1 (5.7)	7.3 (5.4)
	Therapy 4	22.3 (4.4)	6.1 (5.0)	6.5 (5.1)
	Change 1–4	3.7 (4.2) *P* < 0.005	− 5.9 (5.8) *P* < 0.005	− 6.1 (5.3) *P* < 0.005
Participants who completed five sessions (N = 34)	Therapy 1	18.8 (3.5)	13.0 (6.6)	13.1 (4.9)
	Therapy 2	20.2 (4.1)	8.8 (6.7)	9.2 (5.5)
	Therapy 3	21.7 (4.3)	7.2 (6.2)	7.5 (5.4)
	Therapy 4	22.2 (4.0)	6.6 (5.1)	6.6 (5.1)
	Therapy 5	23.4 (5.0)	6.3 (6.3)	6.1 (5.4)
	Change 1–5	4.6 (4.4) *P* < 0.005	− 6.7 (6.3) *P* < 0.005	− 7.0 (5.9) *P* < 0.005

GAD-7 and PHQ-9 scores were not normally distributed or correctable by log transformation at any time point. On visual inspection non-normality was accounted for by extreme outliers. A non-parametric test, Spearman’s Rho was chosen for correlation assessment, allowing assessment of a non-linear relationship. Outliers were adjusted by winsorisation [44] in repeated measures analysis. SWEMWBS scores were normally distributed and no adjustment was applied. Internal consistency calculated using Cronbach’s Alpha was 0.887 at Time 1 (N = 127); and 0.930 at Time 5 (N = 34). Paired samples T-Tests indicated that significant changes in scores were seen from baseline to last measurement across all three measures for participants who completed two, three, four and five sessions respectively.

### Correlation between measures

Correlations between SWEMWBS and PHQ-9 scores were negative and significant (*P* < 0.005) at each time point; coefficients ranged from -0.60 at Therapy 1 to − 0.79 at Therapy 5 (Table 3). Correlations between SWEMWBS scores and GAD-7 scores were also negative and significant (*P* < 0.005) at each time point with coefficients ranging from -0.63 at Therapy 1 to − 0.74 at Therapy 5 (Table 2). Slightly higher positive correlations were found between the two clinical scales, PHQ-9 and GAD-7 (0.73–0.87, *P* < 0.005).

**Table 3:** Correlation of scores on SWEMWBS, PHQ-9 and GAD-7 at all time points

Score	Time point	N	Correlation coefficient	P value (correlation)
SWEMWBS and PHQ-9	Therapy 1	127	− 0.60	< 0.005
	Therapy 2	127	− 0.72	< 0.005
	Therapy 3	94	− 0.67	< 0.005
	Therapy 4	63	− 0.73	< 0.005
	Therapy 5	34	− 0.79	< 0.005
SWEMWBS and GAD-7	Therapy 1	127	− 0.63	< 0.005
	Therapy 2	127	− 0.63	< 0.005
	Therapy 3	94	− 0.65	< 0.005
	Therapy 4	64	− 0.73	< 0.005
	Therapy 5	34	− 0.74	< 0.005
PHQ-9 and GAD-7	Therapy 1	127	0.73	< 0.005
	Therapy 2	127	0.81	< 0.005
	Therapy 3	94	0.83	< 0.005
	Therapy 4	64	0.82	< 0.005
	Therapy 5	34	0.87	< 0.005

### Time series analyses

**Fig. 1:**
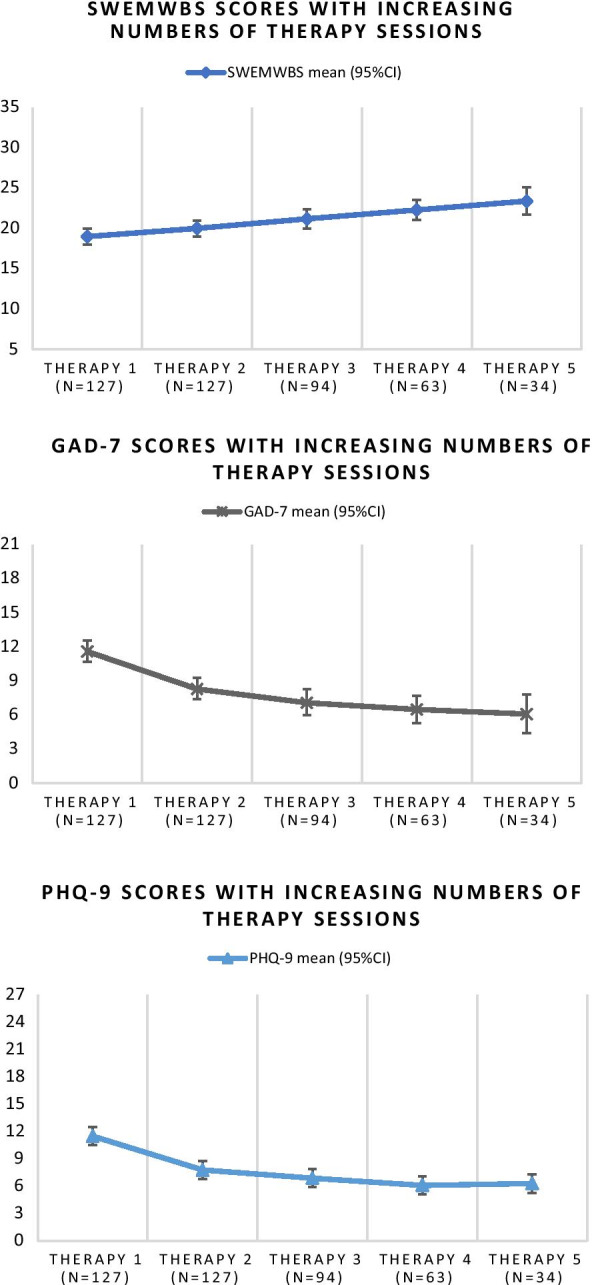
SWEMWBS, PHQ-9 and GAD-7 scores with increasing therapy sessions

Mean SWEMWBS scores increased, and mean PHQ-9 and GAD-7 scores decreased at each consecutive therapy session in all groups. For PHQ-9 and GAD-7, the greatest change in scores was seen between Therapy 1 and Therapy 2, and change tapered over time. SWEMWBS change scores remained consistent between each time point, demonstrating a more linear relationship (see Fig. 1). For each participant group (attending two, three, four and five sessions respectively) a significant (*P* < 0.005) change was seen between baseline and last measurement on all three measures (see Table 4.) For SWEMWBS scores, a linear effect was found for participants who completed three, four and five consecutive sessions, whereas for GAD-7 and PHQ-9 both a linear and a quadratic trend was observed in these groups, consistent with the observed tapering in scores over time.

**Table 4:** Repeated Measures Analyses (GLM repeated measures) of change over time

Participant group (N)	Test	SWEMWBS F statistic (*P*-value)	GAD7 F statistic (*P*-value)	PHQ9 F statistic (*P*-value)
Participants who completed two sessions (N = 127)	Within subject effects	31.760 (< 0.005)	97.205 (< 0.005)	71.041 (< 0.005)
Participants who completed three sessions (N = 94)	Within subject effects	34.094 (< 0.005)*	67.809 (< 0.005)*	54.272 (< 0.005)**
	Within subject contrasts	Linear 45.137 (< 0.005) Quadratic 0.156 (0.690)	Linear 94.756 (< 0.005) Quadratic 6.132 (0.015)**	Linear 76.334 (< 0.005) Quadratic 8.886 (0.004)
Participants who completed four sessions (N = 63)	Within subject effects	33.433 (< 0.005)*	49.312 (< 0.005)**	39.225 (< 0.005)**
	Within subject contrasts	Linear 51.434 (< 0.005) Quadratic 0.234 (0.634)	Linear 83.358 (< 0.005) Quadratic 10.152 (0.002)	Linear 67.198 (< 0.005) Quadratic 11.511 (0.001)
Participants who completed five sessions (N = 34)	Within subject effects	20.294 (< 0.005)*	27.001 (< 0.005)*	19.368 (< 0.005)**
	Within subject contrasts	Linear 38.196 (< 0.005) Quadratic 1.949 (0.172)	Linear 45.44 (< 0.005) Quadratic 25.477 (< 0.005)	Linear 31.234 (< 0.005) Quadratic 21.702 (< 0.005)

^*^Greenhouse Geisser correction applied

^**^Huynh Feldt Correction applied

Total number of sessions completed was also fitted for each time point but this did not have a significant effect at any time point

## Discussion

### Summary of findings

SWEMWBS, PHQ-9 and GAD-7 all showed improvements during treatment and for all three scales there was evidence of individual improvement meeting criteria for statistical and minimally important change at all time points [37, 40, 41]. SWEWMBS scores were strongly and inversely correlated with PHQ-9 and GAD-7 scores at all time points: correlations were stronger at time 5 compared to time 1, increasing with each measurement point (Table 3). The increasing correlations between therapy sessions 1 and 5 suggest that the scales become more convergent as mental health improves for patients. It is not clear why correlations increase: one explanation is that repeated completion of all measures may lead to greater accuracy in recording, a second could be that therapeutic intervention leads to greater self-awareness and thus more precision in completion of measures.

PHQ-9 and GAD-7 scores improved in a curvilinear way with evidence of a quadratic trend in change over time, greatest improvement was seen between Therapy 1 and Therapy 2. SWEMWBS scores also improved but the trend was linear (Table 4). Score distributions on SWEMWBS, but not PHQ-9 or GAD-7 were normally distributed.

The tapering in change scores as participants improved seen with the clinical measures was not evident with SWEMWBS. Results suggest that the two clinical measures show greater change at the lower (less well) end of the mental health spectrum and SWEMWBS may be more sensitive to change at the less unwell end of the spectrum. Differences in the trend of change over time for SWEMWBS and clinical measures and correlation at less than 0.9 at different time points could be due to differences in the measurement properties of the different scales, particularly ceiling effects with the mental illness measures. This suggestive finding needs corroborating in other studies; if it proves valid it would be consistent with the intended purposes of the different scales.

### Implications and comparison to other literature

These results suggest that SWEMWBS shows construct validity as a clinical outcome measure for patients with CMD and can be used to assess change over multiple time points. This, together with patients’ preference for positively focused measures [13], makes SWEMWBS a candidate for evaluating psychological interventions in this setting.

Our findings align with those of a sample of clinical patients with Severe Mental Illness undergoing psychological therapy, where change with similar effect size was seen using WEMWBS when compared to clinical measures [24, 25]. A pilot of a mental health support service for doctors in the UK also found change across both clinical measures and WEMWBS before and after treatment, with a greater effect size seen with WEMWBS, Perceived Stress Scale and Psychological Outcome Profiles (Psychlops) than with PHQ-9 or GAD-7 [45]. This positive focus complements and supports positive psychology and asset-based approaches.

SWEMWBS could be considered either on its own or in conjunction with clinical measures, which may provide greater sensitivity at the onset of intervention when patients have worse levels of mental health. The lack of tapering suggests that SWEMWBS enables measurement beyond clinical cut-off points for CMD and thus meets the need for a measure that is appropriate and valid across public health and clinical interventions. The respective sensitivity of the scales at higher levels of mental health as patients enter recovery phases should be investigated in future research. Given the small sample size and limited diversity of the sample, the findings in this study should be further investigated in larger datasets such as in the NHS where SWEMWBS and WEMWBS are both being used in clinical populations [23]

A recent population-based psychometric analysis [19] of WEMWBS items and items in the psychological distress measure GHQ -12 suggested both scales measure a single dimension or underlying construct. We did not a-priori specify what level of correlation would be sufficient to prove or disprove the dual continuum model for SWEMWBS in comparison to the PHQ-9 and GAD-7 measures of depression and anxiety. Correlations over 0.5 have been hypothesised to show adequate convergent validity [46] in patient reported outcome measures. In a true dual continuum model correlations would be expected to approach 0. The correlations (all > 0.6) we observed in this study suggest at least partial if not full overlap between the constructs.

### Strengths and limitations

Few studies collect data using both positive and negative outcome measures over multiple time points to allow the comparisons made in this study, but the relatively small sample, consisting of mainly white women who were able to pay fees for private therapy, limits conclusions with regard to the changes which might be observed in a more diverse and wider population. Although there is no a priori reason to suppose that the relationship between these scales would be different in different population groups, conclusions drawn would be strengthened by validation in other populations.

Within this cohort the majority of participants completed two sessions. The number of participants with analysable data reduced with each consecutive session. In 46.5% of instances this represented documented planned endings and in others the ending may have been mutually agreed given the nature of the practice. Nonetheless, the possibility remains that the groups completing up to five sessions may have differed to those completing fewer sessions: those who were motivated to attend more sessions may have had a greater capacity to reach higher levels of wellbeing; or those who were not experiencing benefit may have terminated sessions earlier. These limitations would be important if the data were being used to evaluate the effectiveness of CHT, but this was not the purpose of this study. They do not impact the validity of conclusions relating to the measurement properties of the three scales.

Reflection on scores of all three scales during therapy sessions may have introduced social desirability bias but there is no a priori reason to believe that this would have affected responses to the three measures differentially.

## Conclusions

In this exploratory analysis, SWEMWBS demonstrates construct validity and sensitivity to change as a clinical outcome measure for patients with CMDs in primary care, demonstrating inverse correlation and comparable sensitivity to change over a course of clinical treatment when compared to two widely used clinical outcome measures. These analyses provide support for the use of SWEMWBS use as an outcome measure for clinicians and services who wish to respect clients’ preference for SWEMWBS over negative and illness focused measures [18]. Results are compatible with SWEMWBS’ potential as an outcome measure that is valid across clinical and public health settings from treatment to recovery. Given patient preference for positively over negatively framed measures and statistical advantages of measures that are normally distributed, SWEMWBS could be used as an alternative to PHQ-9 and GAD-7 in monitoring and evaluation of CMD treatment. Confirmation of results with larger samples and diverse populations would be valuable.

## Data Availability

Restrictions apply to the availability of these data, which were used under data sharing agreement for the current study, and so are not publicly available. Data are however available from the authors upon reasonable request and with permission of Quest Cognitive Hypnotherapy.

## Abbreviations

WEMWBS: The Warwick-Edinburgh Mental Wellbeing Scale
SWEMWBS: Short Warwick-Edinburgh Mental Wellbeing Scale
CHT: Cognitive hypnotherapy treatment
QCH: Quest cognitive hypnotherapy
PHQ-9: Patient Health Questionnaire-9
GAD-7: General Anxiety Disorder-7
PRIME-MD: Primary Care Evaluation of Mental Disorders
DSM-IV: Diagnostic and Statistical Manual of Mental Disorders, 4th edition
ROC: Receiver operator curve
SD: Standard deviation
CI: Confidence interval
